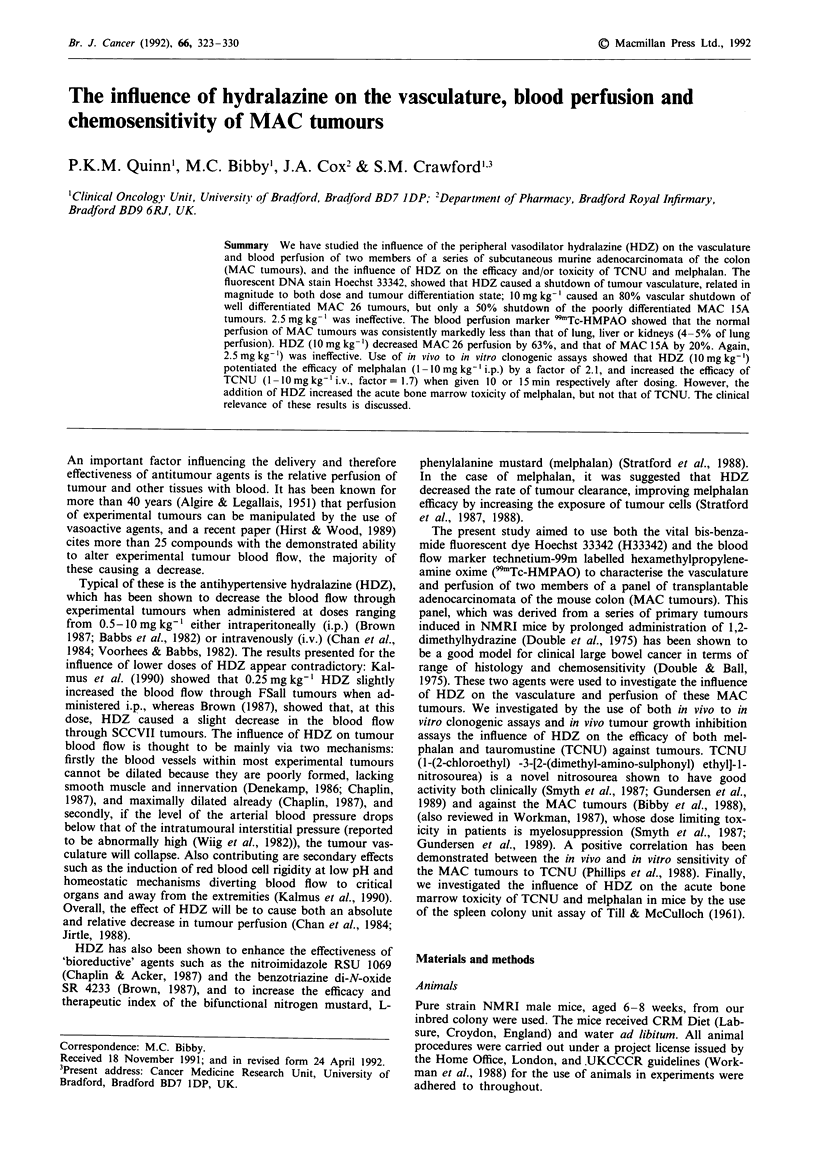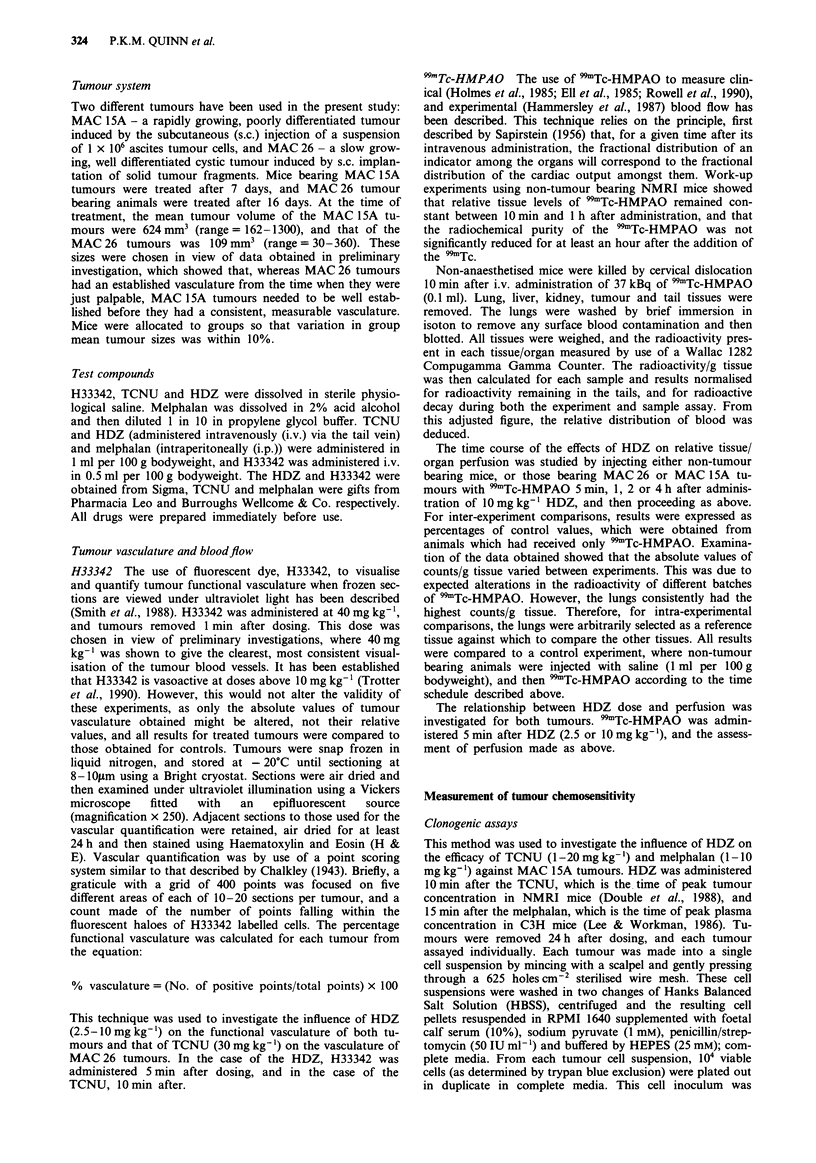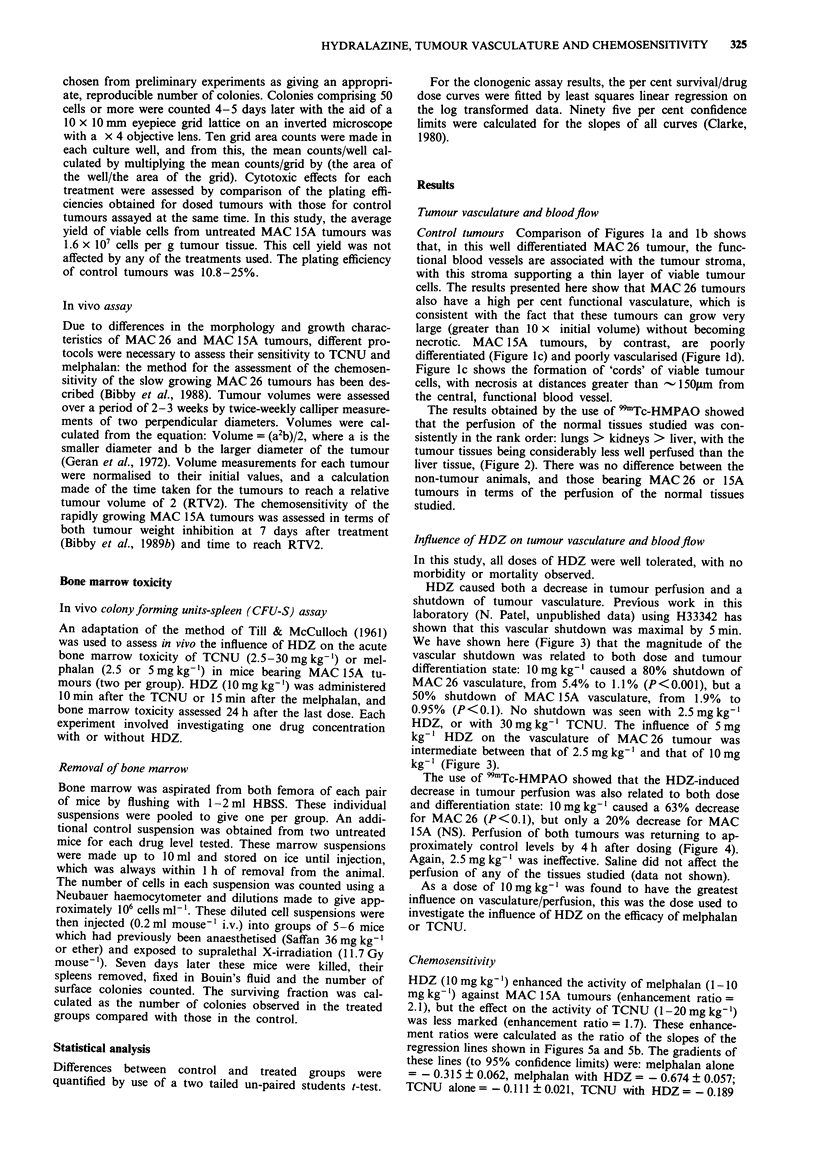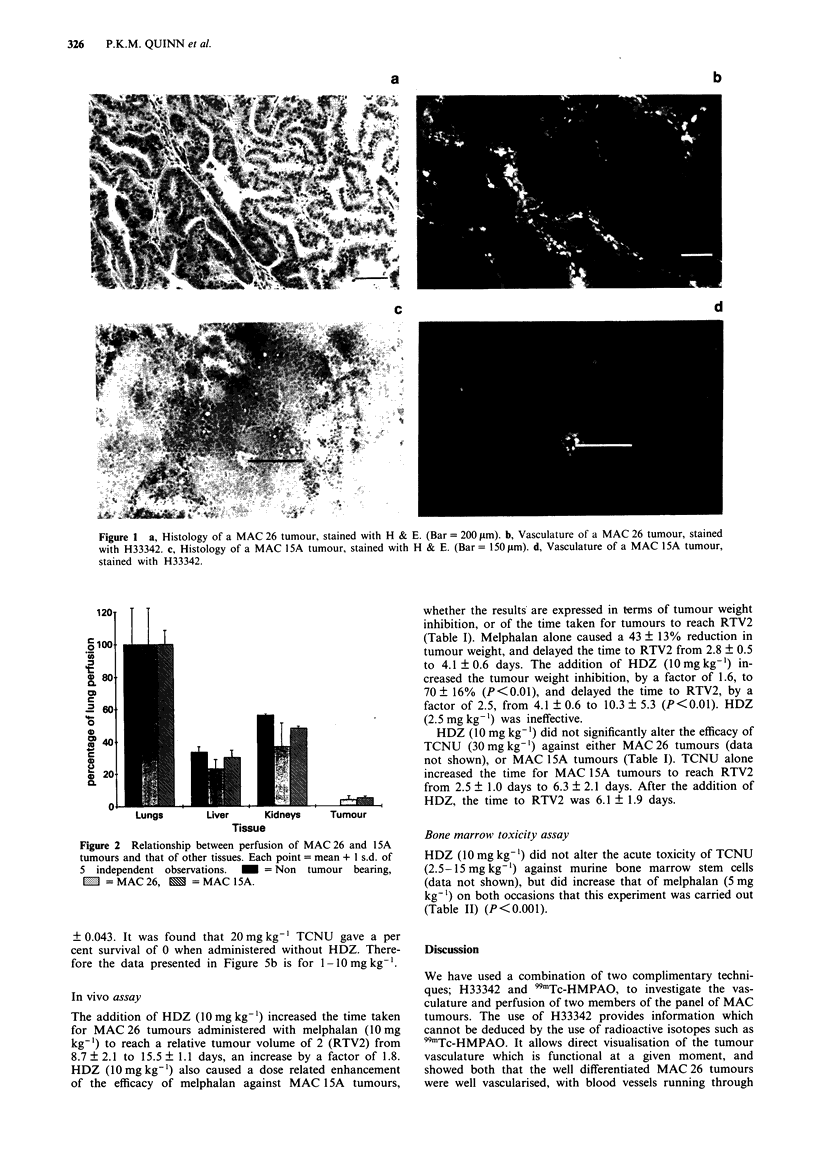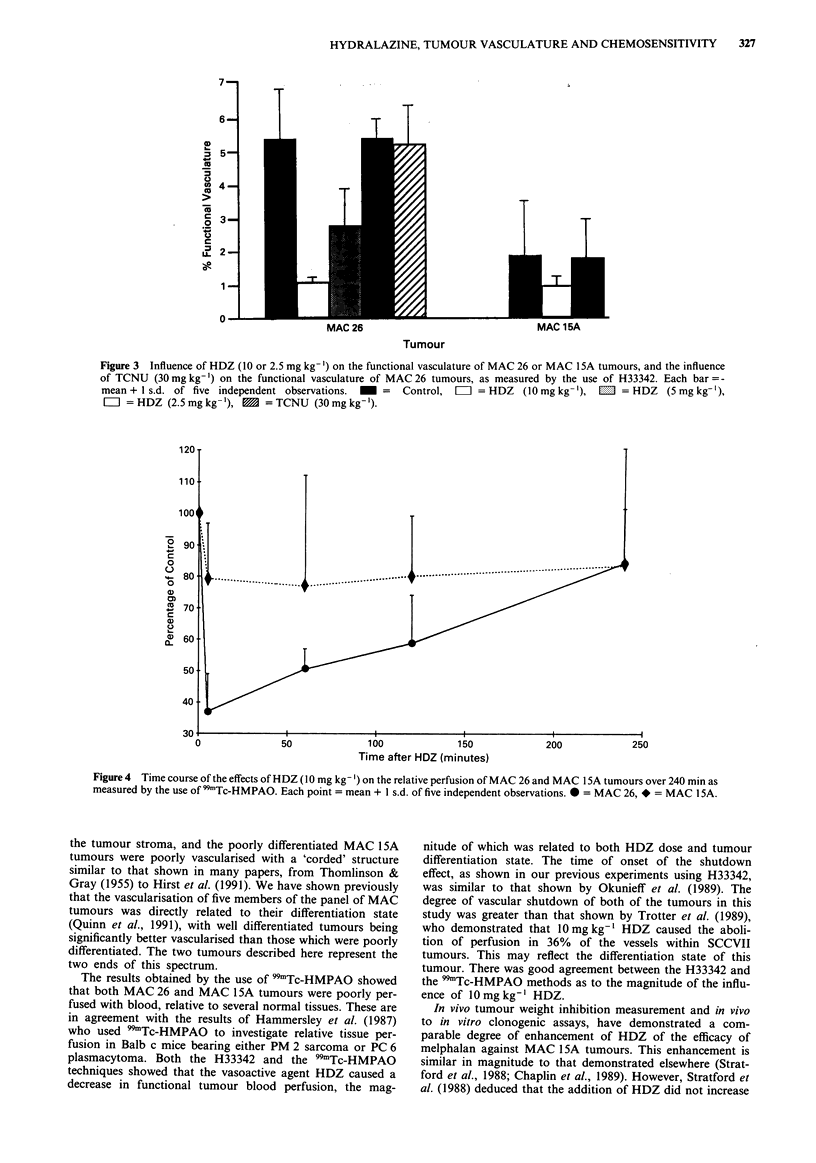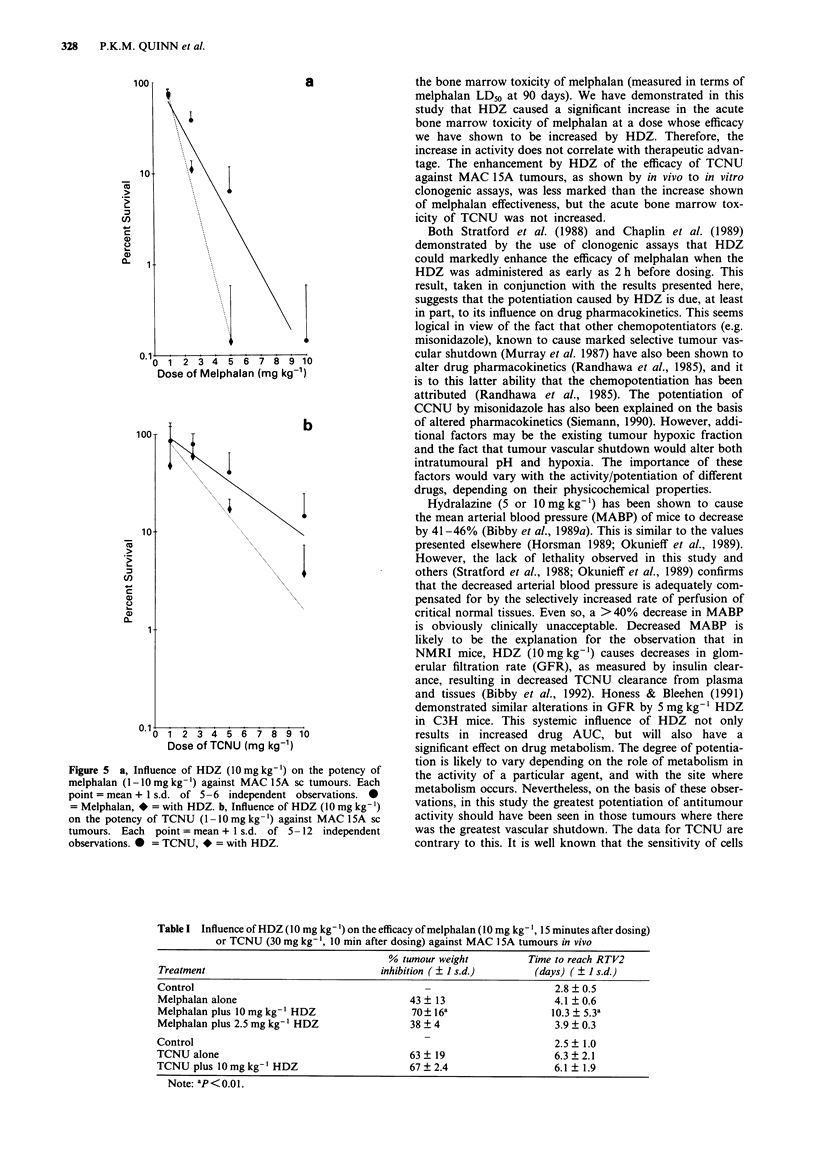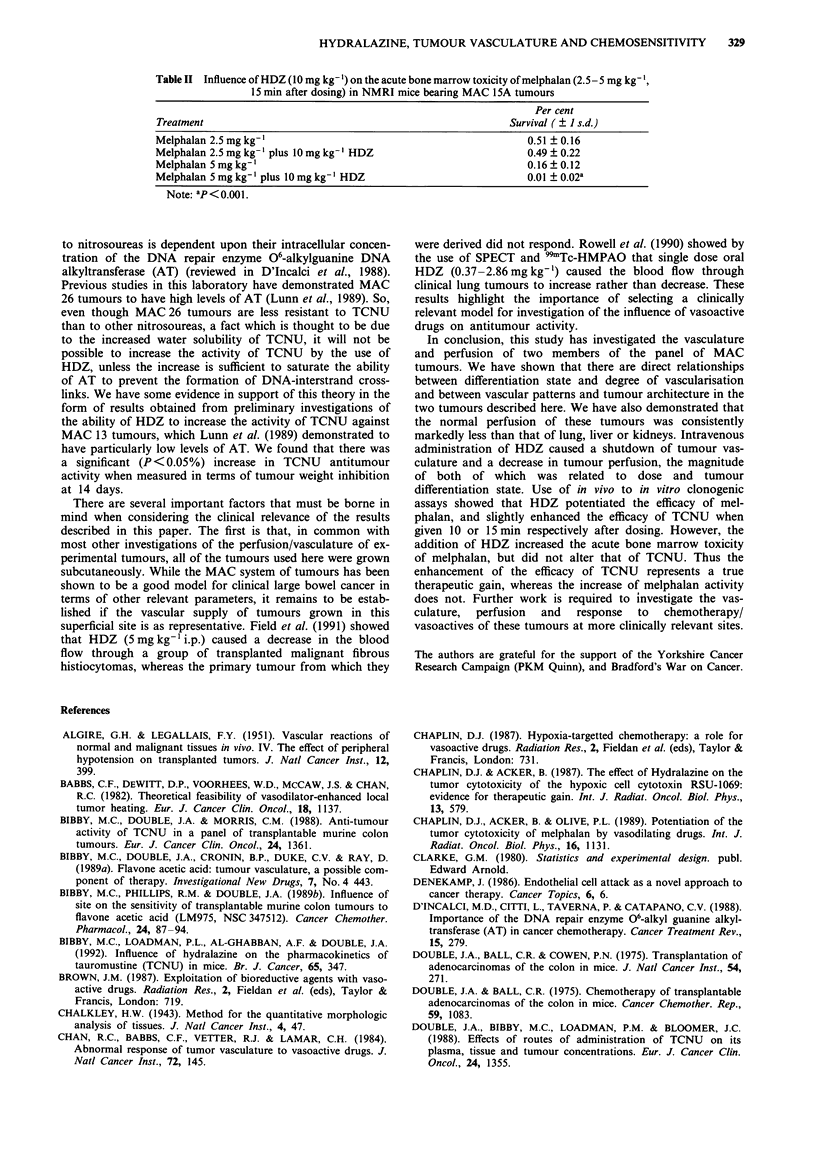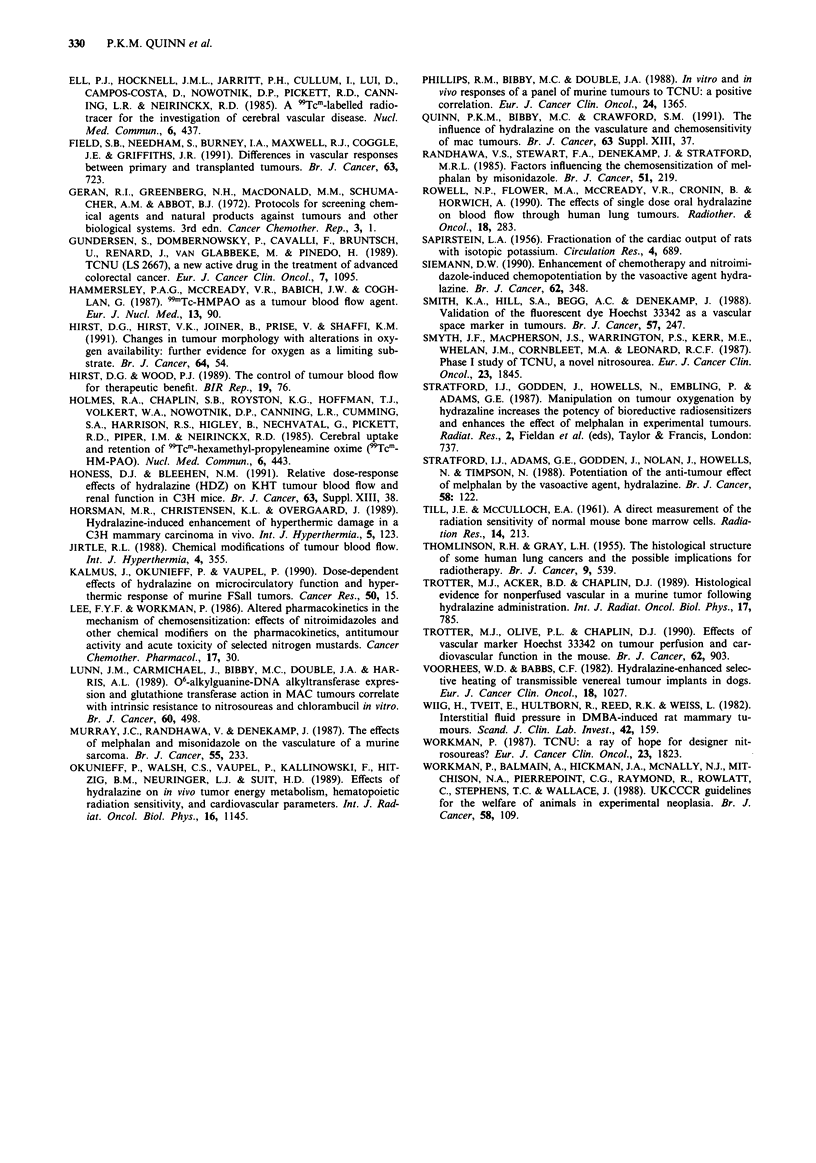# The influence of hydralazine on the vasculature, blood perfusion and chemosensitivity of MAC tumours.

**DOI:** 10.1038/bjc.1992.264

**Published:** 1992-08

**Authors:** P. K. Quinn, M. C. Bibby, J. A. Cox, S. M. Crawford

**Affiliations:** Clinical Oncology Unit, University of Bradford, UK.

## Abstract

**Images:**


Br. J. Cancer (1992), 66, 323-330 © Macmillan Press Ltd., 1992

### The influence of hydralazine on the vasculature, blood perfusion and chemosensitivity of MAC tumours

P.K.M. Quinn[1], M.C. Bibby[1], J.A. Cox[2] & S.M. Crawford[1,3]

[1]*Clinical Oncology Unit, University of Bradford, Bradford BD7 1DP;* [2]*Department of Pharmacy, Bradford Royal Infirmary, Bradford BD9 6RJ, UK.*

**Summary** We have studied the influence of the peripheral vasodilator hydralazine (HDZ) on the vasculature and blood perfusion of two members of a series of subcutaneous murine adenocarcinomata of the colon (MAC tumours), and the influence of HDZ on the efficacy and/or toxicity of TCNU and melphalan. The fluorescent DNA stain Hoechst 33342, showed that HDZ caused a shutdown of tumour vasculature, related in magnitude to both dose and tumour differentiation state; 10 mg kg$^{-1}$ caused an 80% vascular shutdown of well differentiated MAC 26 tumours, but only a 50% shutdown of the poorly differentiated MAC 15A tumours. 2.5 mg kg$^{-1}$ was ineffective. The blood perfusion marker $^{99m}$Tc-HMPAO showed that the normal perfusion of MAC tumours was consistently markedly less than that of lung, liver or kidneys (4–5% of lung perfusion). HDZ (10 mg kg$^{-1}$) decreased MAC 26 perfusion by 63%, and that of MAC 15A by 20%. Again, 2.5 mg kg$^{-1}$) was ineffective. Use of *in vivo* to *in vitro* clonogenic assays showed that HDZ (10 mg kg$^{-1}$) potentiated the efficacy of melphalan (1–10 mg kg$^{-1}$ i.p.) by a factor of 2.1, and increased the efficacy of TCNU (1–10 mg kg$^{-1}$ i.v., factor = 1.7) when given 10 or 15 min respectively after dosing. However, the addition of HDZ increased the acute bone marrow toxicity of melphalan, but not that of TCNU. The clinical relevance of these results is discussed.

An important factor influencing the delivery and therefore effectiveness of antitumour agents is the relative perfusion of tumour and other tissues with blood. It has been known for more than 40 years (Algire & Legallais, 1951) that perfusion of experimental tumours can be manipulated by the use of vasoactive agents, and a recent paper (Hirst & Wood, 1989) cites more than 25 compounds with the demonstrated ability to alter experimental tumour blood flow, the majority of these causing a decrease.

Typical of these is the antihypertensive hydralazine (HDZ), which has been shown to decrease the blood flow through experimental tumours when administered at doses ranging from 0.5–10 mg kg$^{-1}$ either intraperitoneally (i.p.) (Brown 1987; Babbs *et al.*, 1982) or intravenously (i.v.) (Chan *et al.*, 1984; Voorhees & Babbs, 1982). The results presented for the influence of lower doses of HDZ appear contradictory: Kalmus *et al.* (1990) showed that 0.25 mg kg$^{-1}$ HDZ slightly increased the blood flow through FSall tumours when administered i.p., whereas Brown (1987), showed that, at this dose, HDZ caused a slight decrease in the blood flow through SCCVII tumours. The influence of HDZ on tumour blood flow is thought to be mainly via two mechanisms: firstly the blood vessels within most experimental tumours cannot be dilated because they are poorly formed, lacking smooth muscle and innervation (Denekamp, 1986; Chaplin, 1987), and maximally dilated already (Chaplin, 1987), and secondly, if the level of the arterial blood pressure drops below that of the intratumoural interstitial pressure (reported to be abnormally high (Wiig *et al.*, 1982)), the tumour vasculature will collapse. Also contributing are secondary effects such as the induction of red blood cell rigidity at low pH and homeostatic mechanisms diverting blood flow to critical organs and away from the extremities (Kalmus *et al.*, 1990). Overall, the effect of HDZ will be to cause both an absolute and relative decrease in tumour perfusion (Chan *et al.*, 1984; Jirtle, 1988).

HDZ has also been shown to enhance the effectiveness of 'bioreductive' agents such as the nitroimidazole RSU 1069 (Chaplin & Acker, 1987) and the benzotriazine di-*N*-oxide SR 4233 (Brown, 1987), and to increase the efficacy and therapeutic index of the bifunctional nitrogen mustard, L-phenylalanine mustard (melphalan) (Stratford *et al.*, 1988). In the case of melphalan, it was suggested that HDZ decreased the rate of tumour clearance, improving melphalan efficacy by increasing the exposure of tumour cells (Stratford *et al.*, 1987, 1988).

The present study aimed to use both the vital bis-benzamide fluorescent dye Hoechst 33342 (H33342) and the blood flow marker technetium-99m labelled hexamethylpropyleneamine oxime ($^{99m}$Tc-HMPAO) to characterise the vasculature and perfusion of two members of a panel of transplantable adenocarcinomata of the mouse colon (MAC tumours). This panel, which was derived from a series of primary tumours induced in NMRI mice by prolonged administration of 1,2-dimethylhydrazine (Double *et al.*, 1975) has been shown to be a good model for clinical large bowel cancer in terms of range of histology and chemosensitivity (Double & Ball, 1975). These two agents were used to investigate the influence of HDZ on the vasculature and perfusion of these MAC tumours. We investigated by the use of both *in vivo* to *in vitro* clonogenic assays and *in vivo* tumour growth inhibition assays the influence of HDZ on the efficacy of both melphalan and tauromustine (TCNU) against tumours. TCNU (1-(2-chloroethyl) -3-[2-(dimethyl-amino-sulphonyl) ethyl]-1-nitrosourea) is a novel nitrosourea shown to have good activity both clinically (Smyth *et al.*, 1987; Gundersen *et al.*, 1989) and against the MAC tumours (Bibby *et al.*, 1988), (also reviewed in Workman, 1987), whose dose limiting toxicity in patients is myelosuppression (Smyth *et al.*, 1987; Gundersen *et al.*, 1989). A positive correlation has been demonstrated between the *in vivo* and *in vitro* sensitivity of the MAC tumours to TCNU (Phillips *et al.*, 1988). Finally, we investigated the influence of HDZ on the acute bone marrow toxicity of TCNU and melphalan in mice by the use of the spleen colony unit assay of Till & McCulloch (1961).

### Materials and methods

### *Animals*

Pure strain NMRI male mice, aged 6–8 weeks, from our inbred colony were used. The mice received CRM Diet (Labsure, Croydon, England) and water *ad libitum*. All animal procedures were carried out under a project license issued by the Home Office, London, and UKCCCR guidelines (Workman *et al.*, 1988) for the use of animals in experiments were adhered to throughout.

Correspondence: M.C. Bibby.
Received 18 November 1991; and in revised form 24 April 1992.
[3]Present address: Cancer Medicine Research Unit, University of Bradford, Bradford BD7 1DP, UK.

*Tumour system*

Two different tumours have been used in the present study: MAC 15A – a rapidly growing, poorly differentiated tumour induced by the subcutaneous (s.c.) injection of a suspension of $1 \times 10^6$ ascites tumour cells, and MAC 26 – a slow growing, well differentiated cystic tumour induced by s.c. implantation of solid tumour fragments. Mice bearing MAC 15A tumours were treated after 7 days, and MAC 26 tumour bearing animals were treated after 16 days. At the time of treatment, the mean tumour volume of the MAC 15A tumours were 624 $mm^3$ (range = 162–1300), and that of the MAC 26 tumours was 109 $mm^3$ (range = 30–360). These sizes were chosen in view of data obtained in preliminary investigation, which showed that, whereas MAC 26 tumours had an established vasculature from the time when they were just palpable, MAC 15A tumours needed to be well established before they had a consistent, measurable vasculature. Mice were allocated to groups so that variation in group mean tumour sizes was within 10%.

*Test compounds*

H33342, TCNU and HDZ were dissolved in sterile physiological saline. Melphalan was dissolved in 2% acid alcohol and then diluted 1 in 10 in propylene glycol buffer. TCNU and HDZ (administered intravenously (i.v.) via the tail vein) and melphalan (intraperitoneally (i.p.)) were administered in 1 ml per 100 g bodyweight, and H33342 was administered i.v. in 0.5 ml per 100 g bodyweight. The HDZ and H33342 were obtained from Sigma, TCNU and melphalan were gifts from Pharmacia Leo and Burroughs Wellcome & Co. respectively. All drugs were prepared immediately before use.

*Tumour vasculature and blood flow*

*H33342* The use of fluorescent dye, H33342, to visualise and quantify tumour functional vasculature when frozen sections are viewed under ultraviolet light has been described (Smith *et al.*, 1988). H33342 was administered at 40 mg $kg^{-1}$, and tumours removed 1 min after dosing. This dose was chosen in view of preliminary investigations, where 40 mg $kg^{-1}$ was shown to give the clearest, most consistent visualisation of the tumour blood vessels. It has been established that H33342 is vasoactive at doses above 10 mg $kg^{-1}$ (Trotter *et al.*, 1990). However, this would not alter the validity of these experiments, as only the absolute values of tumour vasculature obtained might be altered, not their relative values, and all results for treated tumours were compared to those obtained for controls. Tumours were snap frozen in liquid nitrogen, and stored at – 20°C until sectioning at 8–10μm using a Bright cryostat. Sections were air dried and then examined under ultraviolet illumination using a Vickers microscope fitted with an epifluorescent source (magnification × 250). Adjacent sections to those used for the vascular quantification were retained, air dried for at least 24 h and then stained using Haematoxylin and Eosin (H & E). Vascular quantification was by use of a point scoring system similar to that described by Chalkley (1943). Briefly, a graticule with a grid of 400 points was focused on five different areas of each of 10–20 sections per tumour, and a count made of the number of points falling within the fluorescent haloes of H33342 labelled cells. The percentage functional vasculature was calculated for each tumour from the equation:

$$\% \text{ vasculature} = (\text{No. of positive points/total points}) \times 100$$

This technique was used to investigate the influence of HDZ (2.5–10 mg $kg^{-1}$) on the functional vasculature of both tumours and that of TCNU (30 mg $kg^{-1}$) on the vasculature of MAC 26 tumours. In the case of the HDZ, H33342 was administered 5 min after dosing, and in the case of the TCNU, 10 min after.

*$^{99m}Tc$-HMPAO* The use of $^{99m}$Tc-HMPAO to measure clinical (Holmes *et al.*, 1985; Ell *et al.*, 1985; Rowell *et al.*, 1990), and experimental (Hammersley *et al.*, 1987) blood flow has been described. This technique relies on the principle, first described by Sapirstein (1956) that, for a given time after its intravenous administration, the fractional distribution of an indicator among the organs will correspond to the fractional distribution of the cardiac output amongst them. Work-up experiments using non-tumour bearing NMRI mice showed that relative tissue levels of $^{99m}$Tc-HMPAO remained constant between 10 min and 1 h after administration, and that the radiochemical purity of the $^{99m}$Tc-HMPAO was not significantly reduced for at least an hour after the addition of the $^{99m}$Tc.

Non-anaesthetised mice were killed by cervical dislocation 10 min after i.v. administration of 37 kBq of $^{99m}$Tc-HMPAO (0.1 ml). Lung, liver, kidney, tumour and tail tissues were removed. The lungs were washed by brief immersion in isoton to remove any surface blood contamination and then blotted. All tissues were weighed, and the radioactivity present in each tissue/organ measured by use of a Wallac 1282 Compugamma Gamma Counter. The radioactivity/g tissue was then calculated for each sample and results normalised for radioactivity remaining in the tails, and for radioactive decay during both the experiment and sample assay. From this adjusted figure, the relative distribution of blood was deduced.

The time course of the effects of HDZ on relative tissue/organ perfusion was studied by injecting either non-tumour bearing mice, or those bearing MAC 26 or MAC 15A tumours with $^{99m}$Tc-HMPAO 5 min, 1, 2 or 4 h after administration of 10 mg $kg^{-1}$ HDZ, and then proceeding as above. For inter-experiment comparisons, results were expressed as percentages of control values, which were obtained from animals which had received only $^{99m}$Tc-HMPAO. Examination of the data obtained showed that the absolute values of counts/g tissue varied between experiments. This was due to expected alterations in the radioactivity of different batches of $^{99m}$Tc-HMPAO. However, the lungs consistently had the highest counts/g tissue. Therefore, for intra-experimental comparisons, the lungs were arbitrarily selected as a reference tissue against which to compare the other tissues. All results were compared to a control experiment, where non-tumour bearing animals were injected with saline (1 ml per 100 g bodyweight), and then $^{99m}$Tc-HMPAO according to the time schedule described above.

The relationship between HDZ dose and perfusion was investigated for both tumours. $^{99m}$Tc-HMPAO was administered 5 min after HDZ (2.5 or 10 mg $kg^{-1}$), and the assessment of perfusion made as above.

**Measurement of tumour chemosensitivity**

*Clonogenic assays*

This method was used to investigate the influence of HDZ on the efficacy of TCNU (1–20 mg $kg^{-1}$) and melphalan (1–10 mg $kg^{-1}$) against MAC 15A tumours. HDZ was administered 10 min after the TCNU, which is the time of peak tumour concentration in NMRI mice (Double *et al.*, 1988), and 15 min after the melphalan, which is the time of peak plasma concentration in C3H mice (Lee & Workman, 1986). Tumours were removed 24 h after dosing, and each tumour assayed individually. Each tumour was made into a single cell suspension by mincing with a scalpel and gently pressing through a 625 holes $cm^{-2}$ sterilised wire mesh. These cell suspensions were washed in two changes of Hanks Balanced Salt Solution (HBSS), centrifuged and the resulting cell pellets resuspended in RPMI 1640 supplemented with foetal calf serum (10%), sodium pyruvate (1 mM), penicillin/streptomycin (50 IU $ml^{-1}$) and buffered by HEPES (25 mM); complete media. From each tumour cell suspension, $10^4$ viable cells (as determined by trypan blue exclusion) were plated out in duplicate in complete media. This cell inoculum was chosen from preliminary experiments as giving an appropriate, reproducible number of colonies. Colonies comprising 50 cells or more were counted 4–5 days later with the aid of a 10 × 10 mm eyepiece grid lattice on an inverted microscope with a × 4 objective lens. Ten grid area counts were made in each culture well, and from this, the mean counts/well calculated by multiplying the mean counts/grid by (the area of the well/the area of the grid). Cytotoxic effects for each treatment were assessed by comparison of the plating efficiencies obtained for dosed tumours with those for control tumours assayed at the same time. In this study, the average yield of viable cells from untreated MAC 15A tumours was $1.6 \times 10^7$ cells per g tumour tissue. This cell yield was not affected by any of the treatments used. The plating efficiency of control tumours was 10.8–25%.

### *In vivo assay*

Due to differences in the morphology and growth characteristics of MAC 26 and MAC 15A tumours, different protocols were necessary to assess their sensitivity to TCNU and melphalan: the method for the assessment of the chemosensitivity of the slow growing MAC 26 tumours has been described (Bibby *et al.*, 1988). Tumour volumes were assessed over a period of 2–3 weeks by twice-weekly calliper measurements of two perpendicular diameters. Volumes were calculated from the equation: Volume = $(a^2b)/2$, where a is the smaller diameter and b the larger diameter of the tumour (Geran *et al.*, 1972). Volume measurements for each tumour were normalised to their initial values, and a calculation made of the time taken for the tumours to reach a relative tumour volume of 2 (RTV2). The chemosensitivity of the rapidly growing MAC 15A tumours was assessed in terms of both tumour weight inhibition at 7 days after treatment (Bibby *et al.*, 1989*b*) and time to reach RTV2.

### Bone marrow toxicity

### *In vivo colony forming units-spleen (CFU-S) assay*

An adaptation of the method of Till & McCulloch (1961) was used to assess *in vivo* the influence of HDZ on the acute bone marrow toxicity of TCNU (2.5–30 mg kg$^{-1}$) or melphalan (2.5 or 5 mg kg$^{-1}$) in mice bearing MAC 15A tumours (two per group). HDZ (10 mg kg$^{-1}$) was administered 10 min after the TCNU or 15 min after the melphalan, and bone marrow toxicity assessed 24 h after the last dose. Each experiment involved investigating one drug concentration with or without HDZ.

### *Removal of bone marrow*

Bone marrow was aspirated from both femora of each pair of mice by flushing with 1–2 ml HBSS. These individual suspensions were pooled to give one per group. An additional control suspension was obtained from two untreated mice for each drug level tested. These marrow suspensions were made up to 10 ml and stored on ice until injection, which was always within 1 h of removal from the animal. The number of cells in each suspension was counted using a Neubauer haemocytometer and dilutions made to give approximately $10^6$ cells ml$^{-1}$. These diluted cell suspensions were then injected (0.2 ml mouse$^{-1}$ i.v.) into groups of 5–6 mice which had previously been anaesthetised (Saffan 36 mg kg$^{-1}$ or ether) and exposed to supralethal X-irradiation (11.7 Gy mouse$^{-1}$). Seven days later these mice were killed, their spleens removed, fixed in Bouin's fluid and the number of surface colonies counted. The surviving fraction was calculated as the number of colonies observed in the treated groups compared with those in the control.

### Statistical analysis

Differences between control and treated groups were quantified by use of a two tailed un-paired students *t*-test.

For the clonogenic assay results, the per cent survival/drug dose curves were fitted by least squares linear regression on the log transformed data. Ninety five per cent confidence limits were calculated for the slopes of all curves (Clarke, 1980).

### Results

### *Tumour vasculature and blood flow*

*Control tumours* Comparison of Figures 1a and 1b shows that, in this well differentiated MAC 26 tumour, the functional blood vessels are associated with the tumour stroma, with this stroma supporting a thin layer of viable tumour cells. The results presented here show that MAC 26 tumours also have a high per cent functional vasculature, which is consistent with the fact that these tumours can grow very large (greater than 10 × initial volume) without becoming necrotic. MAC 15A tumours, by contrast, are poorly differentiated (Figure 1c) and poorly vascularised (Figure 1d). Figure 1c shows the formation of 'cords' of viable tumour cells, with necrosis at distances greater than ~150μm from the central, functional blood vessel.

The results obtained by the use of $^{99m}$Tc-HMPAO showed that the perfusion of the normal tissues studied was consistently in the rank order: lungs > kidneys > liver, with the tumour tissues being considerably less well perfused than the liver tissue, (Figure 2). There was no difference between the non-tumour animals, and those bearing MAC 26 or 15A tumours in terms of the perfusion of the normal tissues studied.

### *Influence of HDZ on tumour vasculature and blood flow*

In this study, all doses of HDZ were well tolerated, with no morbidity or mortality observed.

HDZ caused both a decrease in tumour perfusion and a shutdown of tumour vasculature. Previous work in this laboratory (N. Patel, unpublished data) using H33342 has shown that this vascular shutdown was maximal by 5 min. We have shown here (Figure 3) that the magnitude of the vascular shutdown was related to both dose and tumour differentiation state: 10 mg kg$^{-1}$ caused a 80% shutdown of MAC 26 vasculature, from 5.4% to 1.1% ($P < 0.001$), but a 50% shutdown of MAC 15A vasculature, from 1.9% to 0.95% ($P < 0.1$). No shutdown was seen with 2.5 mg kg$^{-1}$ HDZ, or with 30 mg kg$^{-1}$ TCNU. The influence of 5 mg kg$^{-1}$ HDZ on the vasculature of MAC 26 tumour was intermediate between that of 2.5 mg kg$^{-1}$ and that of 10 mg kg$^{-1}$ (Figure 3).

The use of $^{99m}$Tc-HMPAO showed that the HDZ-induced decrease in tumour perfusion was also related to both dose and differentiation state: 10 mg kg$^{-1}$ caused a 63% decrease for MAC 26 ($P < 0.1$), but only a 20% decrease for MAC 15A (NS). Perfusion of both tumours was returning to approximately control levels by 4 h after dosing (Figure 4). Again, 2.5 mg kg$^{-1}$ was ineffective. Saline did not affect the perfusion of any of the tissues studied (data not shown).

As a dose of 10 mg kg$^{-1}$ was found to have the greatest influence on vasculature/perfusion, this was the dose used to investigate the influence of HDZ on the efficacy of melphalan or TCNU.

### *Chemosensitivity*

HDZ (10 mg kg$^{-1}$) enhanced the activity of melphalan (1–10 mg kg$^{-1}$) against MAC 15A tumours (enhancement ratio = 2.1), but the effect on the activity of TCNU (1–20 mg kg$^{-1}$) was less marked (enhancement ratio = 1.7). These enhancement ratios were calculated as the ratio of the slopes of the regression lines shown in Figures 5a and 5b. The gradients of these lines (to 95% confidence limits) were: melphalan alone = $-0.315 \pm 0.062$, melphalan with HDZ = $-0.674 \pm 0.057$; TCNU alone = $-0.111 \pm 0.021$, TCNU with HDZ = $-0.189$

**Figure 1** **a**, Histology of a MAC 26 tumour, stained with H & E. (Bar = 200 μm). **b**, Vasculature of a MAC 26 tumour, stained with H33342. **c**, Histology of a MAC 15A tumour, stained with H & E. (Bar = 150 μm). **d**, Vasculature of a MAC 15A tumour, stained with H33342.

**Figure 2** Relationship between perfusion of MAC 26 and 15A tumours and that of other tissues. Each point = mean + 1 s.d. of 5 independent observations. ■ = Non tumour bearing, ▩ = MAC 26, ▨ = MAC 15A.

$\pm$ 0.043. It was found that 20 mg kg$^{-1}$ TCNU gave a per cent survival of 0 when administered without HDZ. Therefore the data presented in Figure 5b is for 1–10 mg kg$^{-1}$.

### In vivo *assay*

The addition of HDZ (10 mg kg$^{-1}$) increased the time taken for MAC 26 tumours administered with melphalan (10 mg kg$^{-1}$) to reach a relative tumour volume of 2 (RTV2) from 8.7 $\pm$ 2.1 to 15.5 $\pm$ 1.1 days, an increase by a factor of 1.8. HDZ (10 mg kg$^{-1}$) also caused a dose related enhancement of the efficacy of melphalan against MAC 15A tumours, whether the results are expressed in terms of tumour weight inhibition, or of the time taken for tumours to reach RTV2 (Table I). Melphalan alone caused a 43 $\pm$ 13% reduction in tumour weight, and delayed the time to RTV2 from 2.8 $\pm$ 0.5 to 4.1 $\pm$ 0.6 days. The addition of HDZ (10 mg kg$^{-1}$) increased the tumour weight inhibition, by a factor of 1.6, to 70 $\pm$ 16% ($P<0.01$), and delayed the time to RTV2, by a factor of 2.5, from 4.1 $\pm$ 0.6 to 10.3 $\pm$ 5.3 ($P<0.01$). HDZ (2.5 mg kg$^{-1}$) was ineffective.

HDZ (10 mg kg$^{-1}$) did not significantly alter the efficacy of TCNU (30 mg kg$^{-1}$) against either MAC 26 tumours (data not shown), or MAC 15A tumours (Table I). TCNU alone increased the time for MAC 15A tumours to reach RTV2 from 2.5 $\pm$ 1.0 days to 6.3 $\pm$ 2.1 days. After the addition of HDZ, the time to RTV2 was 6.1 $\pm$ 1.9 days.

### *Bone marrow toxicity assay*

HDZ (10 mg kg$^{-1}$) did not alter the acute toxicity of TCNU (2.5–15 mg kg$^{-1}$) against murine bone marrow stem cells (data not shown), but did increase that of melphalan (5 mg kg$^{-1}$) on both occasions that this experiment was carried out (Table II) ($P<0.001$).

### Discussion

We have used a combination of two complimentary techniques; H33342 and $^{99m}$Tc-HMPAO, to investigate the vasculature and perfusion of two members of the panel of MAC tumours. The use of H33342 provides information which cannot be deduced by the use of radioactive isotopes such as $^{99m}$Tc-HMPAO. It allows direct visualisation of the tumour vasculature which is functional at a given moment, and showed both that the well differentiated MAC 26 tumours were well vascularised, with blood vessels running through

**Figure 3** Influence of HDZ (10 or 2.5 mg kg$^{-1}$) on the functional vasculature of MAC 26 or MAC 15A tumours, and the influence of TCNU (30 mg kg$^{-1}$) on the functional vasculature of MAC 26 tumours, as measured by the use of H33342. Each bar = mean + 1 s.d. of five independent observations. ■ = Control, □ = HDZ (10 mg kg$^{-1}$), ▒ = HDZ (5 mg kg$^{-1}$), □ = HDZ (2.5 mg kg$^{-1}$), ▨ = TCNU (30 mg kg$^{-1}$).

**Figure 4** Time course of the effects of HDZ (10 mg kg$^{-1}$) on the relative perfusion of MAC 26 and MAC 15A tumours over 240 min as measured by the use of $^{99m}$Tc-HMPAO. Each point = mean + 1 s.d. of five independent observations. ● = MAC 26, ◆ = MAC 15A.

the tumour stroma, and the poorly differentiated MAC 15A tumours were poorly vascularised with a 'corded' structure similar to that shown in many papers, from Thomlinson & Gray (1955) to Hirst *et al.* (1991). We have shown previously that the vascularisation of five members of the panel of MAC tumours was directly related to their differentiation state (Quinn *et al.*, 1991), with well differentiated tumours being significantly better vascularised than those which were poorly differentiated. The two tumours described here represent the two ends of this spectrum.

The results obtained by the use of $^{99m}$Tc-HMPAO showed that both MAC 26 and MAC 15A tumours were poorly perfused with blood, relative to several normal tissues. These are in agreement with the results of Hammersley *et al.* (1987) who used $^{99m}$Tc-HMPAO to investigate relative tissue perfusion in Balb c mice bearing either PM 2 sarcoma or PC 6 plasmacytoma. Both the H33342 and the $^{99m}$Tc-HMPAO techniques showed that the vasoactive agent HDZ caused a decrease in functional tumour blood perfusion, the magnitude of which was related to both HDZ dose and tumour differentiation state. The time of onset of the shutdown effect, as shown in our previous experiments using H33342, was similar to that shown by Okunieff *et al.* (1989). The degree of vascular shutdown of both of the tumours in this study was greater than that shown by Trotter *et al.* (1989), who demonstrated that 10 mg kg$^{-1}$ HDZ caused the abolition of perfusion in 36% of the vessels within SCCVII tumours. This may reflect the differentiation state of this tumour. There was good agreement between the H33342 and the $^{99m}$Tc-HMPAO methods as to the magnitude of the influence of 10 mg kg$^{-1}$ HDZ.

*In vivo* tumour weight inhibition measurement and *in vivo* to *in vitro* clonogenic assays, have demonstrated a comparable degree of enhancement of HDZ of the efficacy of melphalan against MAC 15A tumours. This enhancement is similar in magnitude to that demonstrated elsewhere (Stratford *et al.*, 1988; Chaplin *et al.*, 1989). However, Stratford *et al.* (1988) deduced that the addition of HDZ did not increase

**Figure 5** **a**, Influence of HDZ (10 mg kg$^{-1}$) on the potency of melphalan (1–10 mg kg$^{-1}$) against MAC 15A sc tumours. Each point = mean + 1 s.d. of 5–6 independent observations. ● = Melphalan, ◆ = with HDZ. **b**, Influence of HDZ (10 mg kg$^{-1}$) on the potency of TCNU (1–10 mg kg$^{-1}$) against MAC 15A sc tumours. Each point = mean + 1 s.d. of 5–12 independent observations. ● = TCNU, ◆ = with HDZ.

the bone marrow toxicity of melphalan (measured in terms of melphalan $LD_{50}$ at 90 days). We have demonstrated in this study that HDZ caused a significant increase in the acute bone marrow toxicity of melphalan at a dose whose efficacy we have shown to be increased by HDZ. Therefore, the increase in activity does not correlate with therapeutic advantage. The enhancement by HDZ of the efficacy of TCNU against MAC 15A tumours, as shown by *in vivo* to *in vitro* clonogenic assays, was less marked than the increase shown of melphalan effectiveness, but the acute bone marrow toxicity of TCNU was not increased.

Both Stratford *et al.* (1988) and Chaplin *et al.* (1989) demonstrated by the use of clonogenic assays that HDZ could markedly enhance the efficacy of melphalan when the HDZ was administered as early as 2 h before dosing. This result, taken in conjunction with the results presented here, suggests that the potentiation caused by HDZ is due, at least in part, to its influence on drug pharmacokinetics. This seems logical in view of the fact that other chemopotentiators (e.g. misonidazole), known to cause marked selective tumour vascular shutdown (Murray *et al.* 1987) have also been shown to alter drug pharmacokinetics (Randhawa *et al.*, 1985), and it is to this latter ability that the chemopotentiation has been attributed (Randhawa *et al.*, 1985). The potentiation of CCNU by misonidazole has also been explained on the basis of altered pharmacokinetics (Siemann, 1990). However, additional factors may be the existing tumour hypoxic fraction and the fact that tumour vascular shutdown would alter both intratumoural pH and hypoxia. The importance of these factors would vary with the activity/potentiation of different drugs, depending on their physicochemical properties.

Hydralazine (5 or 10 mg kg$^{-1}$) has been shown to cause the mean arterial blood pressure (MABP) of mice to decrease by 41–46% (Bibby *et al.*, 1989*a*). This is similar to the values presented elsewhere (Horsman 1989; Okunieff *et al.*, 1989). However, the lack of lethality observed in this study and others (Stratford *et al.*, 1988; Okunieff *et al.*, 1989) confirms that the decreased arterial blood pressure is adequately compensated for by the selectively increased rate of perfusion of critical normal tissues. Even so, a >40% decrease in MABP is obviously clinically unacceptable. Decreased MABP is likely to be the explanation for the observation that in NMRI mice, HDZ (10 mg kg$^{-1}$) causes decreases in glomerular filtration rate (GFR), as measured by insulin clearance, resulting in decreased TCNU clearance from plasma and tissues (Bibby *et al.*, 1992). Honess & Bleehen (1991) demonstrated similar alterations in GFR by 5 mg kg$^{-1}$ HDZ in C3H mice. This systemic influence of HDZ not only results in increased drug AUC, but will also have a significant effect on drug metabolism. The degree of potentiation is likely to vary depending on the role of metabolism in the activity of a particular agent, and with the site where metabolism occurs. Nevertheless, on the basis of these observations, in this study the greatest potentiation of antitumour activity should have been seen in those tumours where there was the greatest vascular shutdown. The data for TCNU are contrary to this. It is well known that the sensitivity of cells

**Table I** Influence of HDZ (10 mg kg$^{-1}$) on the efficacy of melphalan (10 mg kg$^{-1}$, 15 minutes after dosing) or TCNU (30 mg kg$^{-1}$, 10 min after dosing) against MAC 15A tumours *in vivo*

| *Treatment* | *% tumour weight inhibition ( ± 1 s.d.)* | *Time to reach RTV2 (days) ( ± 1 s.d.)* |
|---|---|---|
| Control | – | 2.8 ± 0.5 |
| Melphalan alone | 43 ± 13 | 4.1 ± 0.6 |
| Melphalan plus 10 mg kg$^{-1}$ HDZ | 70 ± 16[a] | 10.3 ± 5.3[a] |
| Melphalan plus 2.5 mg kg$^{-1}$ HDZ | 38 ± 4 | 3.9 ± 0.3 |
| Control | – | 2.5 ± 1.0 |
| TCNU alone | 63 ± 19 | 6.3 ± 2.1 |
| TCNU plus 10 mg kg$^{-1}$ HDZ | 67 ± 2.4 | 6.1 ± 1.9 |

Note: [a]$P < 0.01$.

**Table II** Influence of HDZ (10 mg kg$^{-1}$) on the acute bone marrow toxicity of melphalan (2.5–5 mg kg$^{-1}$, 15 min after dosing) in NMRI mice bearing MAC 15A tumours

| *Treatment* | *Per cent Survival ( ± 1 s.d.)* |
|---|---|
| Melphalan 2.5 mg kg$^{-1}$ | 0.51 ± 0.16 |
| Melphalan 2.5 mg kg$^{-1}$ plus 10 mg kg$^{-1}$ HDZ | 0.49 ± 0.22 |
| Melphalan 5 mg kg$^{-1}$ | 0.16 ± 0.12 |
| Melphalan 5 mg kg$^{-1}$ plus 10 mg kg$^{-1}$ HDZ | 0.01 ± 0.02[a] |

Note: [a]$P<0.001$.

to nitrosoureas is dependent upon their intracellular concentration of the DNA repair enzyme $O^6$-alkylguanine DNA alkyltransferase (AT) (reviewed in D'Incalci *et al.*, 1988). Previous studies in this laboratory have demonstrated MAC 26 tumours to have high levels of AT (Lunn *et al.*, 1989). So, even though MAC 26 tumours are less resistant to TCNU than to other nitrosoureas, a fact which is thought to be due to the increased water solubility of TCNU, it will not be possible to increase the activity of TCNU by the use of HDZ, unless the increase is sufficient to saturate the ability of AT to prevent the formation of DNA-interstrand cross-links. We have some evidence in support of this theory in the form of results obtained from preliminary investigations of the ability of HDZ to increase the activity of TCNU against MAC 13 tumours, which Lunn *et al.* (1989) demonstrated to have particularly low levels of AT. We found that there was a significant ($P<0.05\%$) increase in TCNU antitumour activity when measured in terms of tumour weight inhibition at 14 days.

There are several important factors that must be borne in mind when considering the clinical relevance of the results described in this paper. The first is that, in common with most other investigations of the perfusion/vasculature of experimental tumours, all of the tumours used here were grown subcutaneously. While the MAC system of tumours has been shown to be a good model for clinical large bowel cancer in terms of other relevant parameters, it remains to be established if the vascular supply of tumours grown in this superficial site is as representative. Field *et al.* (1991) showed that HDZ (5 mg kg$^{-1}$ i.p.) caused a decrease in the blood flow through a group of transplanted malignant fibrous histiocytomas, whereas the primary tumour from which they were derived did not respond. Rowell *et al.* (1990) showed by the use of SPECT and $^{99m}$Tc-HMPAO that single dose oral HDZ (0.37–2.86 mg kg$^{-1}$) caused the blood flow through clinical lung tumours to increase rather than decrease. These results highlight the importance of selecting a clinically relevant model for investigation of the influence of vasoactive drugs on antitumour activity.

In conclusion, this study has investigated the vasculature and perfusion of two members of the panel of MAC tumours. We have shown that there are direct relationships between differentiation state and degree of vascularisation and between vascular patterns and tumour architecture in the two tumours described here. We have also demonstrated that the normal perfusion of these tumours was consistently markedly less than that of lung, liver or kidneys. Intravenous administration of HDZ caused a shutdown of tumour vasculature and a decrease in tumour perfusion, the magnitude of both of which was related to dose and tumour differentiation state. Use of *in vivo* to *in vitro* clonogenic assays showed that HDZ potentiated the efficacy of melphalan, and slightly enhanced the efficacy of TCNU when given 10 or 15 min respectively after dosing. However, the addition of HDZ increased the acute bone marrow toxicity of melphalan, but did not alter that of TCNU. Thus the enhancement of the efficacy of TCNU represents a true therapeutic gain, whereas the increase of melphalan activity does not. Further work is required to investigate the vasculature, perfusion and response to chemotherapy/vasoactives of these tumours at more clinically relevant sites.

The authors are grateful for the support of the Yorkshire Cancer Research Campaign (PKM Quinn), and Bradford's War on Cancer.

**References**

ALGIRE, G.H. & LEGALLAIS, F.Y. (1951). Vascular reactions of normal and malignant tissues *in vivo*. IV. The effect of peripheral hypotension on transplanted tumors. *J. Natl Cancer Inst.*, **12,** 399.

BABBS, C.F., DEWITT, D.P., VOORHEES, W.D., MCCAW, J.S. & CHAN, R.C. (1982). Theoretical feasibility of vasodilator-enhanced local tumor heating. *Eur. J. Cancer Clin. Oncol.*, **18,** 1137.

BIBBY, M.C., DOUBLE, J.A. & MORRIS, C.M. (1988). Anti-tumour activity of TCNU in a panel of transplantable murine colon tumours. *Eur. J. Cancer Clin. Oncol.*, **24,** 1361.

BIBBY, M.C., DOUBLE, J.A., CRONIN, B.P., DUKE, C.V. & RAY, D. (1989*a*). Flavone acetic acid: tumour vasculature, a possible component of therapy. *Investigational New Drugs*, **7,** No. 4 443.

BIBBY, M.C., PHILLIPS, R.M. & DOUBLE, J.A. (1989*b*). Influence of site on the sensitivity of transplantable murine colon tumours to flavone acetic acid (LM975, NSC 347512). *Cancer Chemother. Pharmacol.*, **24,** 87–94.

BIBBY, M.C., LOADMAN, P.L., AL-GHABBAN, A.F. & DOUBLE, J.A. (1992). Influence of hydralazine on the pharmacokinetics of tauromustine (TCNU) in mice. *Br. J. Cancer*, **65,** 347.

BROWN, J.M. (1987). Exploitation of bioreductive agents with vasoactive drugs. *Radiation Res.*, **2,** Fieldan *et al.* (eds), Taylor & Francis, London: 719.

CHALKLEY, H.W. (1943). Method for the quantitative morphologic analysis of tissues. *J. Natl Cancer Inst.*, **4,** 47.

CHAN, R.C., BABBS, C.F., VETTER, R.J. & LAMAR, C.H. (1984). Abnormal response of tumor vasculature to vasoactive drugs. *J. Natl Cancer Inst.*, **72,** 145.

CHAPLIN, D.J. (1987). Hypoxia-targetted chemotherapy: a role for vasoactive drugs. *Radiation Res.*, **2,** Fieldan *et al.* (eds), Taylor & Francis, London: 731.

CHAPLIN, D.J. & ACKER, B. (1987). The effect of Hydralazine on the tumor cytotoxicity of the hypoxic cell cytotoxin RSU-1069: evidence for therapeutic gain. *Int. J. Radiat. Oncol. Biol. Phys.*, **13,** 579.

CHAPLIN, D.J., ACKER, B. & OLIVE, P.L. (1989). Potentiation of the tumor cytotoxicity of melphalan by vasodilating drugs. *Int. J. Radiat. Oncol. Biol. Phys.*, **16,** 1131.

CLARKE, G.M. (1980). *Statistics and experimental design*. publ. Edward Arnold.

DENEKAMP, J. (1986). Endothelial cell attack as a novel approach to cancer therapy. *Cancer Topics*, **6,** 6.

D'INCALCI, M.D., CITTI, L., TAVERNA, P. & CATAPANO, C.V. (1988). Importance of the DNA repair enzyme $O^6$-alkyl guanine alkyltransferase (AT) in cancer chemotherapy. *Cancer Treatment Rev.*, **15,** 279.

DOUBLE, J.A., BALL, C.R. & COWEN, P.N. (1975). Transplantation of adenocarcinomas of the colon in mice. *J. Natl Cancer Inst.*, **54,** 271.

DOUBLE, J.A. & BALL, C.R. (1975). Chemotherapy of transplantable adenocarcinomas of the colon in mice. *Cancer Chemother. Rep.*, **59,** 1083.

DOUBLE, J.A., BIBBY, M.C., LOADMAN, P.M. & BLOOMER, J.C. (1988). Effects of routes of administration of TCNU on its plasma, tissue and tumour concentrations. *Eur. J. Cancer Clin. Oncol.*, **24,** 1355.

ELL, P.J., HOCKNELL, J.M.L., JARRITT, P.H., CULLUM, I., LUI, D., CAMPOS-COSTA, D., NOWOTNIK, D.P., PICKETT, R.D., CANNING, L.R. & NEIRINCKX, R.D. (1985). A $^{99}Tc^{m}$-labelled radiotracer for the investigation of cerebral vascular disease. *Nucl. Med. Commun.*, **6**, 437.

FIELD, S.B., NEEDHAM, S., BURNEY, I.A., MAXWELL, R.J., COGGLE, J.E. & GRIFFITHS, J.R. (1991). Differences in vascular responses between primary and transplanted tumours. *Br. J. Cancer*, **63**, 723.

GERAN, R.I., GREENBERG, N.H., MACDONALD, M.M., SCHUMACHER, A.M. & ABBOT, B.J. (1972). Protocols for screening chemical agents and natural products against tumours and other biological systems. 3rd edn. *Cancer Chemother. Rep.*, **3**, 1.

GUNDERSEN, S., DOMBERNOWSKY, P., CAVALLI, F., BRUNTSCH, U., RENARD, J., VAN GLABBEKE, M. & PINEDO, H. (1989). TCNU (LS 2667), a new active drug in the treatment of advanced colorectal cancer. *Eur. J. Cancer Clin. Oncol.*, **7**, 1095.

HAMMERSLEY, P.A.G., MCCREADY, V.R., BABICH, J.W. & COGHLAN, G. (1987). $^{99m}$Tc-HMPAO as a tumour blood flow agent. *Eur. J. Nucl. Med.*, **13**, 90.

HIRST, D.G., HIRST, V.K., JOINER, B., PRISE, V. & SHAFFI, K.M. (1991). Changes in tumour morphology with alterations in oxygen availability: further evidence for oxygen as a limiting substrate. *Br. J. Cancer*, **64**, 54.

HIRST, D.G. & WOOD, P.J. (1989). The control of tumour blood flow for therapeutic benefit. *BIR Rep.*, **19**, 76.

HOLMES, R.A., CHAPLIN, S.B., ROYSTON, K.G., HOFFMAN, T.J., VOLKERT, W.A., NOWOTNIK, D.P., CANNING, L.R., CUMMING, S.A., HARRISON, R.S., HIGLEY, B., NECHVATAL, G., PICKETT, R.D., PIPER, I.M. & NEIRINCKX, R.D. (1985). Cerebral uptake and retention of $^{99}Tc^{m}$-hexamethyl-propyleneamine oxime ($^{99}Tc^{m}$-HM-PAO). *Nucl. Med. Commun.*, **6**, 443.

HONESS, D.J. & BLEEHEN, N.M. (1991). Relative dose-response effects of hydralazine (HDZ) on KHT tumour blood flow and renal function in C3H mice. *Br. J. Cancer*, **63**, Suppl. XIII, 38.

HORSMAN, M.R., CHRISTENSEN, K.L. & OVERGAARD, J. (1989). Hydralazine-induced enhancement of hyperthermic damage in a C3H mammary carcinoma in vivo. *Int. J. Hyperthermia.*, **5**, 123.

JIRTLE, R.L. (1988). Chemical modifications of tumour blood flow. *Int. J. Hyperthermia*, **4**, 355.

KALMUS, J., OKUNIEFF, P. & VAUPEL, P. (1990). Dose-dependent effects of hydralazine on microcirculatory function and hyperthermic response of murine FSall tumors. *Cancer Res.*, **50**, 15.

LEE, F.Y.F. & WORKMAN, P. (1986). Altered pharmacokinetics in the mechanism of chemosensitization: effects of nitroimidazoles and other chemical modifiers on the pharmacokinetics, antitumour activity and acute toxicity of selected nitrogen mustards. *Cancer Chemother. Pharmacol.*, **17**, 30.

LUNN, J.M., CARMICHAEL, J., BIBBY, M.C., DOUBLE, J.A. & HARRIS, A.L. (1989). $O^6$-alkylguanine-DNA alkyltransferase expression and glutathione transferase action in MAC tumours correlate with intrinsic resistance to nitrosoureas and chlorambucil *in vitro*. *Br. J. Cancer*, **60**, 498.

MURRAY, J.C., RANDHAWA, V. & DENEKAMP, J. (1987). The effects of melphalan and misonidazole on the vasculature of a murine sarcoma. *Br. J. Cancer*, **55**, 233.

OKUNIEFF, P., WALSH, C.S., VAUPEL, P., KALLINOWSKI, F., HITZIG, B.M., NEURINGER, L.J. & SUIT, H.D. (1989). Effects of hydralazine on *in vivo* tumor energy metabolism, hematopoietic radiation sensitivity, and cardiovascular parameters. *Int. J. Radiat. Oncol. Biol. Phys.*, **16**, 1145.

PHILLIPS, R.M., BIBBY, M.C. & DOUBLE, J.A. (1988). *In vitro* and *in vivo* responses of a panel of murine tumours to TCNU: a positive correlation. *Eur. J. Cancer Clin. Oncol.*, **24**, 1365.

QUINN, P.K.M., BIBBY, M.C. & CRAWFORD, S.M. (1991). The influence of hydralazine on the vasculature and chemosensitivity of mac tumours. *Br. J. Cancer*, **63** Suppl. XIII, 37.

RANDHAWA, V.S., STEWART, F.A., DENEKAMP, J. & STRATFORD, M.R.L. (1985). Factors influencing the chemosensitization of melphalan by misonidazole. *Br. J. Cancer*, **51**, 219.

ROWELL, N.P., FLOWER, M.A., MCCREADY, V.R., CRONIN, B. & HORWICH, A. (1990). The effects of single dose oral hydralazine on blood flow through human lung tumours. *Radiother. & Oncol.*, **18**, 283.

SAPIRSTEIN, L.A. (1956). Fractionation of the cardiac output of rats with isotopic potassium. *Circulation Res.*, **4**, 689.

SIEMANN, D.W. (1990). Enhancement of chemotherapy and nitroimidazole-induced chemopotentiation by the vasoactive agent hydralazine. *Br. J. Cancer*, **62**, 348.

SMITH, K.A., HILL, S.A., BEGG, A.C. & DENEKAMP, J. (1988). Validation of the fluorescent dye Hoechst 33342 as a vascular space marker in tumours. *Br. J. Cancer*, **57**, 247.

SMYTH, J.F., MACPHERSON, J.S., WARRINGTON, P.S., KERR, M.E., WHELAN, J.M., CORNBLEET, M.A. & LEONARD, R.C.F. (1987). Phase I study of TCNU, a novel nitrosourea. *Eur. J. Cancer Clin. Oncol.*, **23**, 1845.

STRATFORD, I.J., GODDEN, J., HOWELLS, N., EMBLING, P. & ADAMS, G.E. (1987). Manipulation on tumour oxygenation by hydrazaline increases the potency of bioreductive radiosensitizers and enhances the effect of melphalan in experimental tumours. *Radiat. Res.*, **2**, Fieldan *et al.* (eds), Taylor & Francis, London: 737.

STRATFORD, I.J., ADAMS, G.E., GODDEN, J., NOLAN, J., HOWELLS, N. & TIMPSON, N. (1988). Potentiation of the anti-tumour effect of melphalan by the vasoactive agent, hydralazine. *Br. J. Cancer*, **58**: 122.

TILL, J.E. & MCCULLOCH, E.A. (1961). A direct measurement of the radiation sensitivity of normal mouse bone marrow cells. *Radiation Res.*, **14**, 213.

THOMLINSON, R.H. & GRAY, L.H. (1955). The histological structure of some human lung cancers and the possible implications for radiotherapy. *Br. J. Cancer*, **9**, 539.

TROTTER, M.J., ACKER, B.D. & CHAPLIN, D.J. (1989). Histological evidence for nonperfused vascular in a murine tumor following hydralazine administration. *Int. J. Radiat. Oncol. Biol. Phys.*, **17**, 785.

TROTTER, M.J., OLIVE, P.L. & CHAPLIN, D.J. (1990). Effects of vascular marker Hoechst 33342 on tumour perfusion and cardiovascular function in the mouse. *Br. J. Cancer*, **62**, 903.

VOORHEES, W.D. & BABBS, C.F. (1982). Hydralazine-enhanced selective heating of transmissible venereal tumour implants in dogs. *Eur. J. Cancer Clin. Oncol.*, **18**, 1027.

WIIG, H., TVEIT, E., HULTBORN, R., REED, R.K. & WEISS, L. (1982). Interstitial fluid pressure in DMBA-induced rat mammary tumours. *Scand. J. Clin. Lab. Invest.*, **42**, 159.

WORKMAN, P. (1987). TCNU: a ray of hope for designer nitrosoureas? *Eur. J. Cancer Clin. Oncol.*, **23**, 1823.

WORKMAN, P., BALMAIN, A., HICKMAN, J.A., MCNALLY, N.J., MITCHISON, N.A., PIERREPOINT, C.G., RAYMOND, R., ROWLATT, C., STEPHENS, T.C. & WALLACE, J. (1988). UKCCCR guidelines for the welfare of animals in experimental neoplasia. *Br. J. Cancer*, **58**, 109.